# The Mitochondrial Genomes of *Nuttalliella namaqua* (Ixodoidea: Nuttalliellidae) and *Argas africolumbae* (Ixodoidae: Argasidae): Estimation of Divergence Dates for the Major Tick Lineages and Reconstruction of Ancestral Blood-Feeding Characters

**DOI:** 10.1371/journal.pone.0049461

**Published:** 2012-11-08

**Authors:** Ben J. Mans, Daniel de Klerk, Ronel Pienaar, Minique H. de Castro, Abdalla A. Latif

**Affiliations:** 1 Parasites, Vectors and Vector-borne Diseases, Agricultural Research Council-Onderstepoort Veterinary Institute, Onderstepoort, South Africa; 2 The Department of Veterinary Tropical Diseases, University of Pretoria, Pretoria, South Africa; 3 The Biotechnology Platform, Agricultural Research Council-Onderstepoort Veterinary Institute, Onderstepoort, South Africa; Universidade Federal do Rio de Janeiro, Brazil

## Abstract

Ixodida are composed of hard (Ixodidae), soft (Argasidae) and the monotypic Nuttalliellidae (*Nuttalliella namaqua*) tick families. Nuclear 18S rRNA analysis suggested that *N. namaqua* was the closest extant relative to the last common ancestral tick lineage. The mitochondrial genomes of *N. namaqua* and *Argas africolumbae* were determined using next generation sequencing and *de novo* assembly to investigate this further. The latter was included since previous estimates on the divergence times of argasids lacked data for this major genus. Mitochondrial gene order for both was identical to that of the Argasidae and Prostriata. Bayesian analysis of the COI, Cytb, ND1, ND2 and ND4 genes confirmed the monophyly of ticks, the basal position of *N. namaqua* to the other tick families and the accepted systematic relationships of the other tick genera. Molecular clock estimates were derived for the divergence of the major tick lineages and supported previous estimates on the origins of ticks in the Carboniferous. *N. namaqua* larvae fed successfully on lizards and mice in a prolonged manner similar to many argasids and all ixodids. Excess blood meal-derived water was secreted via the salivary glands, similar to ixodids. We propose that this prolonged larval feeding style eventually gave rise to the long feeding periods that typify the single larval, nymphal and adult stages of ixodid ticks and the associated secretion of water via the salivary glands. Ancestral reconstruction of characters involved in blood-feeding indicates that most of the characteristics unique to either hard or soft tick families were present in the ancestral tick lineage.

## Introduction

The Ixodida (ticks) are composed of three families, Argasidae (soft ticks ∼200 species), Ixodidae (hard ticks ∼700 species) and the monotypic Nuttalliellidae [1]–[2].

Differentiation of the Argasidae and Ixodidae is uncomplicated based on biology and morphology. Hard ticks of all life stages possess a sclerotized scutum, an apical located gnathostoma, feed for prolonged periods (several days to weeks) and ingest more than 100× their body mass in blood [3]–[4]. Soft ticks do not possess a scutum, their prognathous mouthparts are located anterior ventrally and they have a leathery integument that can rapidly expand, allowing nymphs and adults to engorge up to ten times their body mass within minutes to hours [3]–[4]. Hard ticks secrete blood meal-derived water back into the host via their salivary glands, while soft ticks use their coxal organs [3]–[4]. *Nuttalliella namaqua*, however, exhibit features associated with both hard and soft ticks. Like soft ticks, its nymphal and adult stages possess a leathery cuticle and engorge rapidly [5]. However, larvae possess a sclerotized scutum, while nymphs and adults have a semi-sclerotized pseudo-scutum and their mouthparts are located apically [5]–[8]. It differs from the other families in that nymphal and adult stages possess ball and socket leg joints and blood meal-derived water is secreted via the Malpighian tubules [5]–[7]. Classification with regard to its relationship to the other tick families based on morphology and biology therefore remains problematic [1].

Analysis of the nuclear 18S ribosomal RNA from *N. namaqua* indicated that it grouped basal to the other tick families, suggesting that it is the closest extant relative to the last common ancestral tick lineage [5]. The monophyly of the Ixodida as well the validity of the Nuttalliellidae as a separate tick family [1]–[2], [9], were also supported. The monophyly of the main tick families was also supported by analysis of mitochondrial genomes [10]. However, discrepancies between nuclear and mitochondrial gene phylogenies have been found within arthropods and ticks [11]–[14]. This raises the question whether mitochondrial data will also support the position of *N. namaqua* at the base of the tick tree. Divergence dates were also estimated for the origin of ticks, based on the mitochondrial dataset [10], but did not include data for *N. namaqua*, or the genus *Argas*. The date estimates for the Ixodida as well as the Argasidae therefore lacked important divergence points, one for the origin of the Ixodida and the second for the divergence of the main argasid lineages. To address these issues, we sequenced the mitochondrial genomes of *N. namaqua* and *Argas africolumbae* [15]. We also derived parameters associated with feeding of larval *N. namaqua* that we used in ancestral reconstruction. A model for the evolution of characters involved in blood-feeding were presented and resolved in part the existing dichotomy regarding the biology of the two major families [4].

## Results and Discussion

### Comparison of mitochondrial genomes

The mitochondrial genome gene order found in *Nuttalliella* and *Argas* was similar to argasids (Ornithodorinae), prostriate ixodids (*Ixodes*) and the inferred ancestral gene order of all arthropods as exhibited by *Limulus polyphemus* [16]–[18]. This included 13 protein-coding genes, the 16S and 12S ribosomal RNA genes, 22 tRNA genes and a single control region (Table 1). Given the basal position of *N. namaqua* to the other tick families [5], this suggested that the ancestral arthropod gene order was conserved in the last common ancestral tick lineage. This gene order differed from that of the metastriate ticks where a block of genes (ND1-tRNA-Glu) has translocated [14], [16]–[17]. Prediction of the secondary structures of the tRNA genes indicated that they can form the conventional four-leaved clover structures, except for the tRNA's for serine that lack the D-loop, similar to other metazoans [18].

**Table 1:** The mitochondrial genomes of *N. namaqua* and *A. africolumbae* compared to *C. capensis*, *O. moubata and I. hexagonus* [18].

	*N. namaqua* (14431 bp)			*A. africolumbae* (14440 bp)			*C. capensis* (14418 bp)			*O. moubata* (14398 bp)			*I. hexagonus* (14539 bp)		
Gene	Position	Size	Size	Position	Size	Size	Position	Size	Size	Position	Size	Size	Position	Size	Size
		Nt	AA		Nt	AA		Nt	AA		Nt	AA		Nt	AA
tRNA-Met	1–67	67		1–66	66		1–60	60		1–60	60		1–65	65	
ND2	67–1017	951	316	66–1022	957	318	62–1017	956	318	62–1023	962	320	82–1038	957	318
tRNA-Trp	1018–1080	63		1020–1083	64		1018–1078	61		1024–1083	60		1039–1103	65	
tRNA-Cys*	1080–1141	62		1075–1139	65		1072–1135	64		1077–1138	62		1097–1156	60	
tRNA-Tyr*	1145–1207	63		1138–1201	64		1139–1197	59		1139–1198	60		1163–1224	62	
COI	1202–2737	1536	511	1193–2731	1539	512	1191–2729	1539	512	1192–2730	1539	512	1218–2756	1539	512
COII	2742–3416	676	225	2739–3413	676	225	2733–3408	676	225	2739–3414	676	225	2760–3435	676	225
tRNA-Lys	3414–3483	70		3414–3482	69		3409–3474	66		3415–3482	58		3436–3504	69	
tRNA-Asp	3480–3542	63		3479–3544	66		3474–3531	58		3482–3539	58		3505–3566	62	
ATP8	3551–3694	144	47	3544–3699	156	51	3533–3688	156	51	3541–3696	156	51	3569–3724	156	51
ATP6	3688–4347	660	219	3693–4361	669	222	3682–4350	669	222	3690–4358	669	222	3721–4383	663	220
COIII	4347–5132	786	261	4361–5140	781	260	4350–5129	780	259	4358–5135	778	259	4383–5166	784	261
tRNA-Gly	5137–5197	61		5141–5204	64		5130–5190	61		5136–5196	61		5168–5231	64	
ND3	5195–5533	339	112	5204–5548	345	114	5191–5526	336	111	5197–5530	334	111	5233–5568	336	111
tRNA-Ala	5532–5594	63		5543–5602	60		5527–5586	60		5531–5592	60		5570–5631	62	
tRNA-Arg	5593–5653	61		5603–5661	59		5587–5643	57		5593–5652	60		5632–5690	59	
tRNA-Asn	5655–5717	63		5659–5720	62		5645–5705	61		5654–5715	62		5691–5756	66	
tRNA-Ser (GCT)	5715–5766	52		5718–5770	53		5708–5760	53		5713–5765	53		5760–5813	54	
tRNA-Glu	5766–5832	67		5770–5831	62		5761–5820	60		5766–5825	60		5815–5875	61	
tRNA-Phe*	5828–5897	70		5828–5889	62		5819–5877	59		5824–5882	59		5874–5933	60	
ND5*	5871–7553	1683	560	5863–7551	1689	562	5878–7537	1660	553	5883–7543	1661	553	5940–7602	1663	554
tRNA-His*	7554–7635	82		7552–7612	61		7538–7598	61		7544–7604	61		7604–7669	66	
ND4*	7636–8955	1320	439	7613–8938	1326	441	7599–8914	1316	438	7605–8925	1321	440	7679–8989	1311	436
ND4L*	8960–9238	279	92	8932–9210	279	92	8908–9186	279	92	8919–9197	279	92	8995–9270	276	91
tRNA-Thr	9243–9305	63		9219–9281	63		9194–9253	60		9203–9262	60		9273–9336	64	
tRNA-Pro*	9303–9367	65		9281–9344	64		9254–9314	61		9263–9322	60		9337–9397	61	
ND6	9369–9800	432	143	9347–9778	432	143	9317–9745	429	142	9325–9759	435	144	9406–9831	426	141
Cyt b	9800–10876	1077	358	9778–10881	1104	367	9751–10851	1101	366	9759–10858	1100	366	9831–10929	1099	366
tRNA-Ser (TGA)	10877–10947	71		10880–10943	64		10852–10914	63		10859–10919	61		10912–10978	67	
ND1*	10937–11881	945	314	10898–11881	985	328	10858–11835	978	325	10873–11847	975	324	10976–11915	940	313
tRNA-Leu (TTR)*	11882–11949	68		11879–11938	60		11839–11899	61		11851–11909	59		11917–11980	64	
tRNA-Leu (TAG)*	11950–12013	64		11941–12003	63		11902–11964	63		11914–11973	60		11990–12050	61	
16S-RNA*	12014–13177	1164		12004–13201	1198		11965–13189	1225		11974–13185	1212		12051–13337	1287	
tRNA-Val*	13178–13235	58		13202–13262	61		13190–13254	65		13186–13243	58		13279–13337	59	
12S-rRNA*	13236–13968	733		13263–13957	695		13255–13949	695		13244–13929	686		13344–14048	705	
Control region	13969–14307	339		13958–14300	342		13950–14291	342		13930–14271	342		14049–14407	359	
tRNA-Ile	14308–14371	64		14301–14368	68		14292–14354	63		14272–14335	64		14408–14471	64	
tRNA-Gln*	14370–5	67		14371–14439	69		14352–14417	63		14333–1	67		14471–14536	66	

Genes occurring on the complementary strands are labelled with an asterisk. Also indicated are the size of the genes, the size of the proteins encoded by the genes and the position on the mitochondrial genome.

### Phylogenetic analysis using the 16S rRNA gene

**Figure 1:**
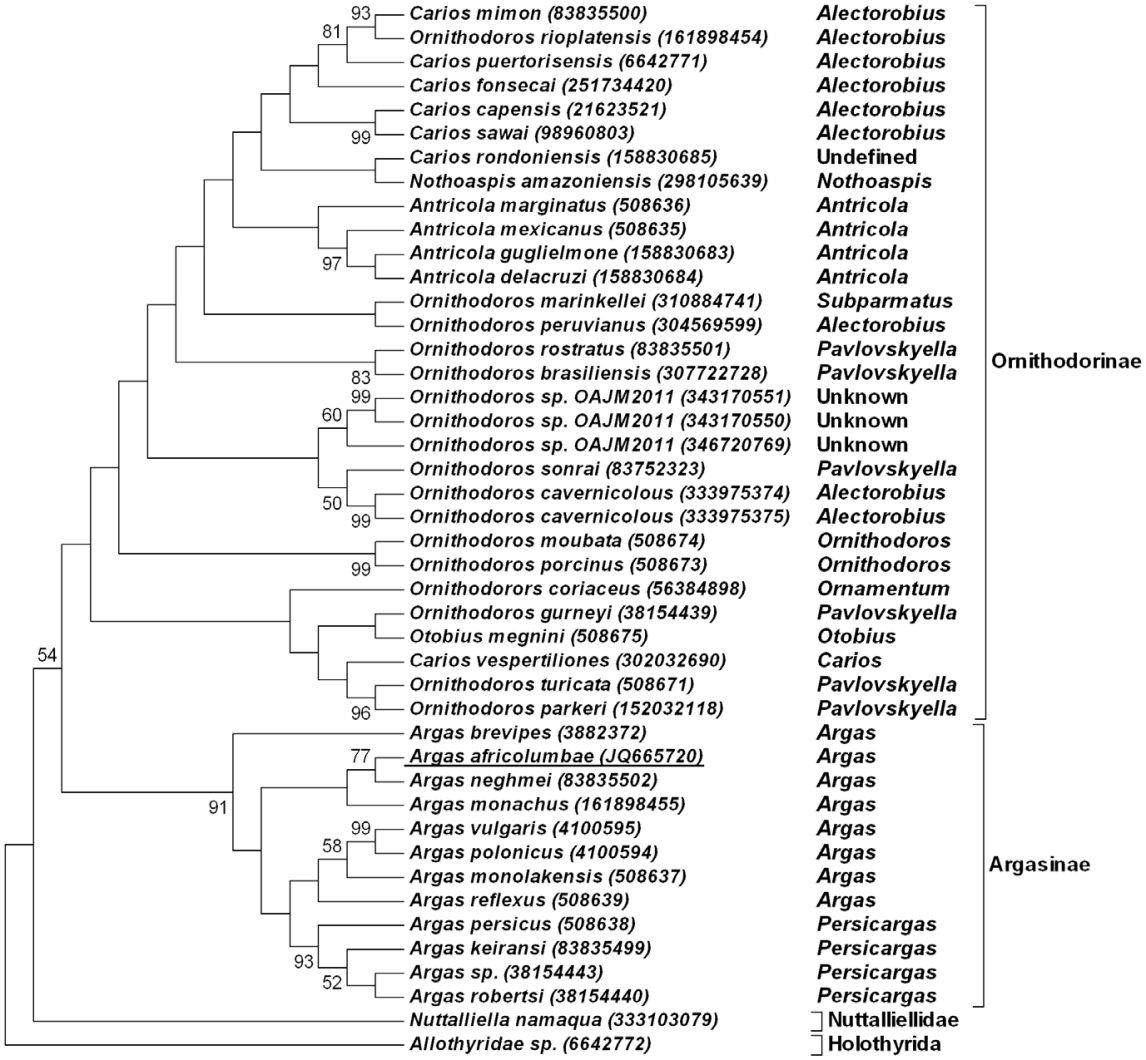
Maximum parsimony analysis using the 16S rRNA gene. The 50% consensus tree from 10000 bootstraps with support above 50% is shown. Species are indicated by their species designation in their Genbank description lines with accession numbers in parenthesis. New sequences reported in this study are underlined. Sub-genera as described by Hoogstraal are indicated [21].

Given the current confusion in argasid systematics [19]–[20], all argasid species will be referred to according to the sub-genera classification system of Hoogstraal [21]. The use of the 16S rRNA gene predominates in argasid molecular systematics, even though recognized to be of little use for resolution of relationships above genus or sub-genus level [19]–[20]. However, it is useful to identify closely related species or groups. BLASTN analysis using the 16S rRNA gene for *A. (Argas) africolumbae* retrieved as best hits members of the genus *Argas* (6E^−164^-1E^−110^) in agreement with its original classification [15]. Phylogenetic analysis indicated that *A. (Argas) africolumbae* grouped with members of the genus *Argas*, with *A. (Argas) neghmei* as closest neighbour (Fig. 1). Nodal support was overall weak confirming previous observations [19]–[20]. The rest of the tree includes the Ornithodorinae which shows little support for any of the classic taxonomical classifications proposed [21]–[23], as reviewed recently [20].

### Phylogenetic analysis using the 18S rRNA gene

**Figure 2:**
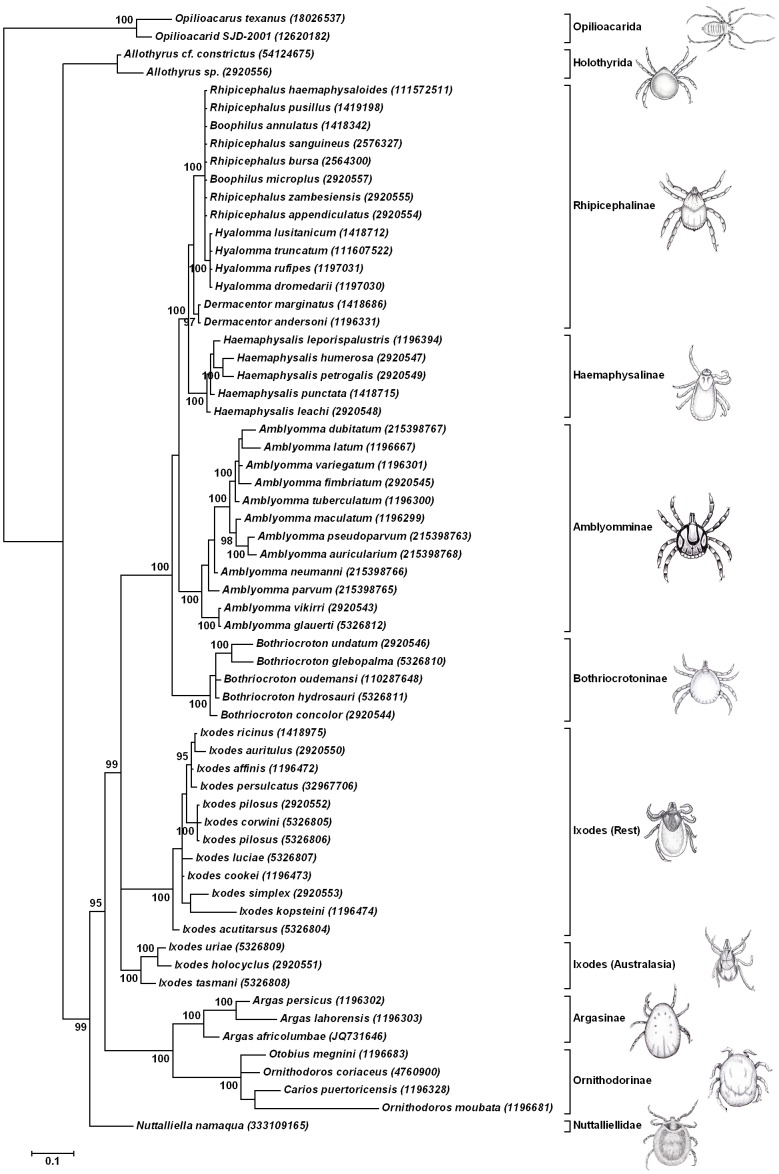
Bayesian analysis using the 18S rRNA gene. The 50% consensus tree is shown with posterior probability support for nodes higher than 90% indicated. Nodes were condensed below 90%. The scale bar indicates Bayesian distances. Species are designated by their Genbank description lines and accession numbers in parenthesis.

To confirm the relationship of *A. (Argas) africolumbae* in the genus *Argas* and retest the hypothesis that *N. namaqua* group at the base of the tick tree, a phylogenetic analysis using the 18S rRNA gene was performed using Bayesian analysis. For this, all available tick 18S rRNA sequences >1500bp was included as well as Opilioacarid and Holothyrid sequences for outgroups (Fig. 2). *A. (Argas) africolumbae* grouped basal in a monophyletic clade with *A.* (*Persicargas*) *persicus* and *A.* (*Alveonasus*) *lahorensis* supporting the monophyly of the genus *Argas* [22]–[23]. As with the 16S rRNA analysis, the 18S rRNA dataset did not resolve members of the Ornithodorinae. However, the monophyly of ticks, the basal position of *N. namaqua* to the argasid and ixodid families and established relationships of most major tick genera as previously defined by 18S rRNA analysis were overall well supported [1], [9].

### Phylogenetic analysis using mitochondrial genomic data

**Figure 3:**
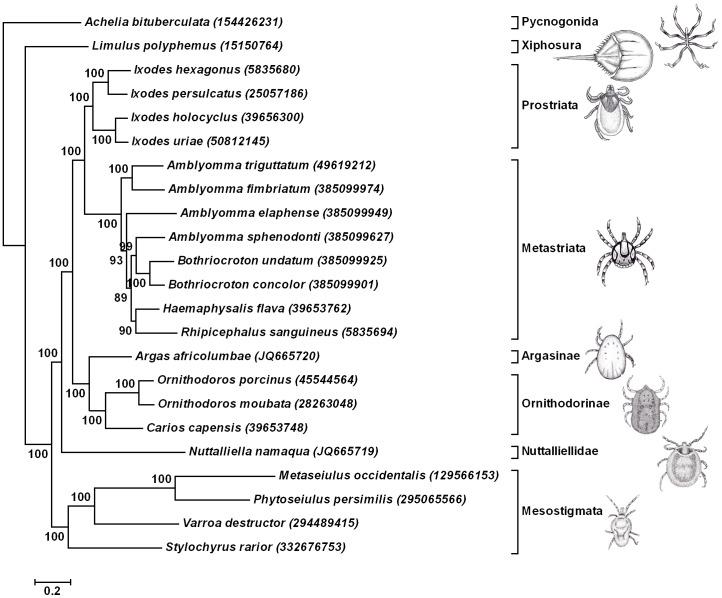
Bayesian analysis using the mitochondrial protein gene set (COI-Cytb-ND1- ND2-ND4). The 50% consensus tree is shown with nodal posterior probability support above 90% indicated. Species are designated by their Genbank description lines and accession numbers in parenthesis. The scale bar indicates Bayesian distances.

Both 16S and 18S data do not resolve all nodes in their respective trees or give nodes with low support. This might be due to low taxon sampling, saturation of phylogenetic signal or inappropriate mutation time scales for the markers used. They are as such not useful for use in molecular divergence estimation. Mitochondrial genome data was investigated as an alternative marker since this was previously used for molecular divergence estimation [10]. Nucleotide compositional reverse strand-bias exists in arthropod mitochondrial genomes and can lead to artificial clustering during phylogenetic analysis [24]. The existence of mutational saturation, especially at deep levels of phylogeny also makes the use of protein sequences more attractive than nucleotide sequences [25]. A recent in-depth analysis of the mitochondrial genes from insects showed that not all mitochondrial genes were suitable for analysis of systematic relationships at deep phylogenetic levels, specifically due to long-branch attraction [25]. The genes most suitable to retrieve the correct topology included the cytochrome c oxidase I (COI), cytochrome b (Cytb), NADH dehydrogenase 1, 2 and 4 (ND1, ND2 and ND4) genes [25]. To test the hypotheses that ticks are monophyletic and that *N. namaqua* groups basal to the tick families, a concatenated supermatrix of the COI, Cytb, ND1, ND2 and ND4 protein genes were analysed using Bayesian analysis. The horseshoe crab, *L. polyphemus* and the pycnogonid, *Achelia bituberculata* were used as outgroups according to the Euchelicerate hypothesis [26]. The majority of the nodes for the 50% consensus tree gave posterior probabilities above 95% (Fig. 3), which were considered to be significant [27]. Within the Parasitiformes, *N. namaqua* grouped at the base of the Ixodida supporting the monophyly of ticks in agreement with previous analysis based on the nuclear 18S rRNA gene [5]. The branch for the hard and soft tick families retrieved recognized relationships for the tick families and genera, with the Argasinae and Ornithodorinae forming a monophyletic clade [1]. Relationships within the Metastriata did not correlate with nuclear 18S rRNA data, similar to recent findings based on mitochondrial data [14].

### Estimation of divergence dates for the major tick lineages

The topology obtained using Bayesian analysis (Fig. 3) was used for the estimation of divergence dates (Table 2). Divergence times were estimated for the Ixodida-Mesostigmata (350±23 MYA), Ixodida (319±25 MYA), the Argasidae-Ixodidae (290±23 MYA) and the Prostriate-Metastriates (249±23 MYA). Within the Argasidae the molecular divergence between the Argasinae and Ornithodorinae was estimated at 234±25 MYA and between the subgenus *Ornithodoros* and *Carios (Alectorobius) capensis* at 158±24 MYA. However, as indicated by cladistic as well as 16S analysis, a close genetic relationship exists between *Alectorobius*, *Antricola* and *Nothoaspis* [19]–[23] and these sub-genera were also included in this split.

**Table 2:** Estimation of the divergence dates for the major lineages of the Ixodida.

Node	Fossil record (MYA)	Mean±SD	Lower-Upper limit
Xiphosura-Pycnogonida	445	444±3	440–449
Ixodida-Mesostigmata		350±23	299–392
Mesostigmata		318±29	257–370
Ixodida		319±25	268–365
Argasidae-Ixodidae		290±23	245–334
Ixodidae		249±23	206–295
*Ixodes* (Australia)-*Ixodes* (Rest)		217±24	171–266
Metastriate	100	124±17	101–166
Argasinae-Ornithodorinae		234±25	184–285
*Ornithodoros*-*Carios*	94	158±24	111–210

Indicated are oldest fossil records for divergent lineages [36]–[38], [44], [66].

### Implications of molecular clock estimates for the divergence of the major tick lineages

**Figure 4:**
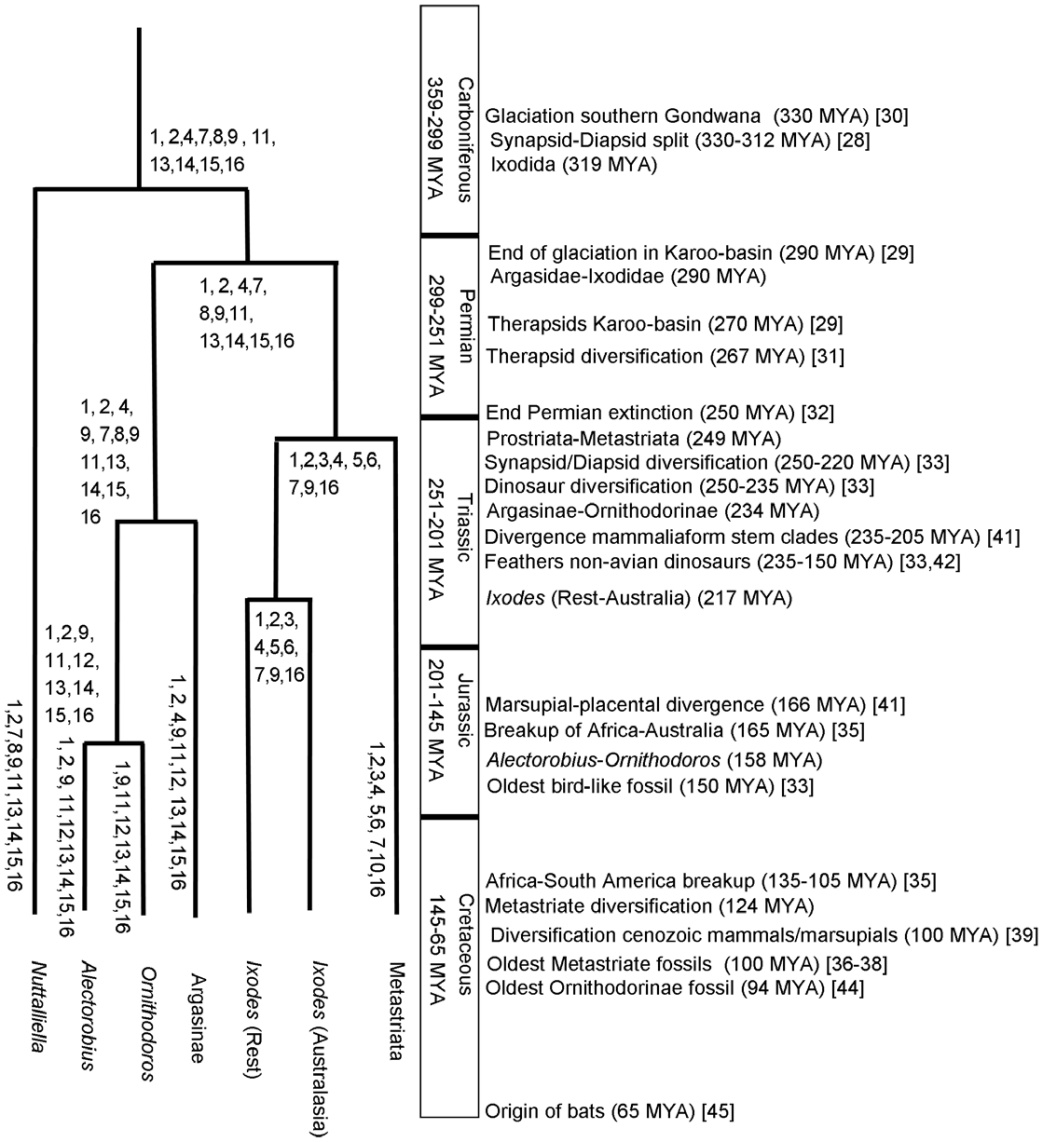
A model for the evolution of blood-feeding characteristics in the Ixodida. Indicated are the date estimates for various nodes of the Ixodida and corresponding major events in evolution. Dates and names for Periods were obtained from the Geologic Time Scale [67]. Blood feeding characters proposed to be present in ancestral and extant lineages are indicated by numbers at the different nodes and include: 1) Haller's organ for host detection. 2) Prolonged feeding in larvae and secretion of water via the salivary glands. 3) Prolonged feeding in nymphs and adults with water secretion via the salivary glands. 4) Rapid digestion of blood meal with lysis of red blood cells during engorgement. 5) One larval and nymphal molt. 6) Adult stages feed once and lay one large egg batch. 7) Presence of scutum. 8) Presence of pseudo-scutum. 9) Little or no cement. 10) Cement. 11) Fast feeding in nymphs and adults with water secretion via the Malpighian tubules. 12) Fast feeding in nymphs and adults with water secretion via the coxal organs. 13) Slow digestion of blood meal with little initial red blood cell lysis. 14) One larval and several nymphal molting stages. 15) Adult stages feed more than once and lay several small egg batches. 16) Loss of heme synthesizing capability and mechanisms to deal with heme toxicity.

Molecular clock estimates placed the origin of ticks in the Carboniferous (Fig. 4), coinciding with the divergence of diapsids and synapsids [28]. The estimates corresponded to previous estimates based on mitochondrial genome analysis [10], but were earlier than the proposed origins for the Ixodida in the middle Permian before the Late Permian extinction [5]. The Permian hypothesis placed the origin of ticks in the Karoo basin in southern Africa and coincided with the origins of therapsids in this area [5], [29]. However, due to extensive glaciation of southern Gondwana (330–290 MYA) that only ended in the early Permian [29]–[30], and the limited distribution of *N. namaqua* in Africa [5], an origin in the Carboniferous would have had to occur in Northern Gondwana, most probably the region that now constitute Eastern Africa. This scenario implies subsequent migration and establishment of *N. namaqua* in the Karoo basin in the late Permian [29].

Hard and soft ticks diverged during the Early Permian and coincided with the origin and diversification of therapsids [29]–[31]. Prostriate-metastriate divergence occurred after the End Permian extinction event and coincided with the diversification of dinosaurs, diapsids and synapsids [32]–[33]. The Prostriates can be divided into *Ixodes* from Australasia and the rest of the World, which grouped into specific clades that suggested geographical isolation [34]. Molecular estimates for the divergence of the different *Ixodes* lineages occur well before the breakup of Gondwana into landmasses that separated Africa and Australia [35]. The divergence of the metastriates correspond to the oldest ixodid fossils found to date, *Cornupalpatum burmanicum*, *Compluriscutula vetulum* and *Amblyomma* larvae, which were found in Burmese amber (100 MYA) [36]–[38]. These fossil species are morphologically the closest to the genus *Amblyomma* and therefore the root of the metastriate lineage. Divergence of the metastriates also coincided with the radiation of cenozoic mammals and marsupials [39]. Within the metastriates, the genus *Bothriocroton* was considered basal and only occurred in Australia and this has been used as evidence for an origin of ticks as well as metastriate ticks in Australasia [1], [40]. The estimates for the diversification of the metastriates and the breakup of Africa and Australia seem to support this. It should, however, be considered that Australia's link with Africa was via Antarctica, a large landmass that showed a rich fauna and flora before glaciation 35 MYA [35]. South America was also linked to the Antarctic Peninsula up to 35 MYA [35]. The origins of the Australian *Ixodes* lineage and the metastriate ticks could therefore have occurred in this area and colonization of the rest of the world by the metastriate ticks could have occurred via South America. It should also be noted that recent analysis of mitochondrial genomes of *Bothriocroton* placed doubt on its designation as a basal metastriate lineage [14], and by implication on the origins of ticks in the Australasian region.

Speciation of the Argasinae-Ornithodorinae occurred at a time when non-avian dinosaur lineages evolved feather-like appendages and when the most basal mammaliaform stem clades where diverging [41]–[42]. This could be significant since the main *Argas* sub-genera (*Argas* and *Persicargas*) are exclusive parasites of birds and their body plans are flat to enable movement through feathers, while most Ornithodorinae feed on mammals [19]–[23], [43]. Adaptation to the avian lineage would have been due to this ecological niche that opened up, rather than the much later evolution of flight [33], [42].

Estimates for the *Ornithodoros*-*Alectorobius* split predate the oldest argasid fossil, *Carios jerseyi*, identified in New Jersey amber (94 MYA) [44]. The *Ornithodoros*-*Alectorobius* split occurred ∼30 million years before the breakup of Africa and South America [35]. It is likely that several basal sub-genera (i.e. *Pavlovskyella*, *Reticulinasus*) diverged in this period, which would put the divergence of the *Alectorobius*-*Antricola* lineage close to the Africa-South America breakup. This would fit with the majority of *Alectorobius* and all *Antricola* being endemic to the New World (Neotropics and Nearctic), while those species that do occur in the rest of the world parasitize marine birds [19]–[23].

It is of interest, that the majority of ticks from the subgenera *Alectorobius, Antricola, Carios, Chiropterargas, Nothoaspis, Parantricola, Reticulinasus* and *Subparmatus* parasitize bats [19]–[23], since the current molecular clock estimates would place the origin of these lineages above 100 MYA. Bats only originated in the Early Paleocene (∼65 MYA) [45] which would suggest that all of these lineages adapted to bats independently, and is supported by paraphyly of *Alectorobius-Antricola* and *Carios* as suggested by 16S rRNA analysis. A possible common denominator in this would be that the ancestral Ornithodorinae parasitized burrow-inhabiting mammals that frequented cave systems and that the roosting behaviour of bats predisposed them towards parasitism by argasids. The peculiar biology of *Antricola* and *Nothoaspis* (adults are not obligate blood-feeding parasites, but possibly feed on bat guano) [20], would therefore be a recent derived character from within the *Alectorobius* lineage.

### Biological data for ancestral character re-construction

#### Larval feeding of *N. namaqua*

The biology of *N. namaqua* was further investigated to obtain information that can be used for ancestral character reconstruction. To this end, it was important to determine whether larvae feed in a prolonged manner like ixodids and some argasids, since this might provide clues to the large biological differences observed between hard and soft ticks [4]. Since no data were available for the feeding kinetics of *N. namaqua* larvae, both lizards and mice were infested with recently hatched larvae. Larvae were obtained from two female ticks that each laid ∼150 eggs from which larvae hatched after ∼14 days [8]. For lizards and mice, 9 and 50 larvae, respectively, fed to engorgement and molted to nymphs. In the case of the lizards the mean attachment time was 8–9 days and for the mice 4–5 days. In both cases a final rapid engorgement phase was observed on the last day of attachment. Approximately an eighty fold increase in body volume occurred during engorgement. Feeding of larvae on mice injected with bromophenol blue indicated that the salivary glands showed coloration, i.e. uptake of bromophenol blue, while the Malpighian tubules remained white. Similar results were obtained when it was shown that the coxal organs were responsible for water secretion in argasids [46], indicating active transport of bromophenol blue into these organs. Secretion of bromophenol blue stained saliva was also observed in a larvae that were detached prior to final engorgement. It was therefore concluded that the most likely route for blood meal concentration, is secretion of water back into the host, in a similar manner to ixodid ticks [3]–[4].

#### Multiple feeding events and storage of blood meal

The association of *N. namaqua* with a variety of hosts as revealed by gut meal analysis [5], raised questions regarding the ability to store blood meal over prolonged time periods, the maintenance of blood meal content during molting between life stages and whether ticks could feed multiple times. As proof of principle studies, intact red blood cells could be detected in the gut meal content of recently molted nymphs of which the larvae had fed on lizards (nucleated red blood cells) and mice (non-nucleated red blood cells), as well as an adult tick that fed more than six months previously. Female ticks that laid eggs, subsequently fed successfully and laid another egg batch. It was concluded that female *N. namaqua* could feed multiple times between egg laying cycles, stored blood meal for prolonged periods in an intact form and retained intact red blood cells after molting.

### Reconstruction of the evolution of characters involved in blood-feeding behaviour

Phylogenetic analysis using the nuclear 18S rRNA gene and mitochondrial protein genes confirmed the grouping of *N. namaqua* at the base of the tick tree. This correlates with morphological and biological observations of different life stages of *N. namaqua* that indicate the presence of various features associated with both hard and soft ticks. It also assists in the evolutionary reconstruction of various characteristics associated with blood-feeding behaviour and the biology of ticks (Fig. 4), as well as a model for the differences observed between the hard and soft tick families [4].

Features that can be reconstructed as being present in ancestral tick lineages include (Fig. 4):

1) Haller's organ for host detection. Since Haller's organ is also present in the holothyrids [9], it was probably co-opted for host detection in the ancestral tick lineage.

2–3) Prolonged larval, nymphal and female feeding, with a rapid engorgement phase that results in blood meal intake >10-fold of body mass, accompanied by secretion of excess blood meal-derived water via the salivary glands. This feeding mode has been retained in larvae of *N. namaqua* and some argasid lineages (*Argas, Alveonasus, Pavlovskyella, Alectorobius, Antricola*), as well as all life stages of ixodids [21], [43], [47]. In the subgenus *Ornithodoros*, larvae do not feed but molt directly to nymphs and should be considered a derived character [21], [43]. We postulate that secretion of excess water via the salivary glands occurs in argasid larvae, since the latter unlike adults do not possess coxal organs [48]. This is therefore an ancestral trait of larval feeding that was retained in nymphal and adult ixodid ticks.

4) Rapid lysis of red blood cells during prolonged feeding would allow for maximal concentration of the blood meal. This ability is present in all ixodids as well as some argasid larvae [47], although it was not specifically observed in *N. namaqua* larvae.

5–6) The ability to take in an excessively large blood meal and process it while feeding, probably led to subsequent changes in ixodid lifestyle that included a single nymphal molt and only one feeding event in the female before laying a large egg batch.

7–8) The presence of a scutum may be associated with the “ixodid-like” morphology of the larval ticks in the ancestral lineage that was retained by ixodids of all life stages. In this regard, the scutum serves a protective role against host grooming during the long attachment periods of feeding [3], [47]. It also explains the presence of the pseudo-scutum in the nymphs and adults of *N. namaqua*. In this regard, the scutum was considered to be ancestral since it is also present in other mites [49].

9–10) Prolonged feeding led to ixodids developing the ability to anchor themselves mechanically in the hosts dermis using cement [3], [47]. In prostriates, which have long mouthparts this ability is limited, while it is well developed in those metastriates with short mouthparts [3], [47], [50]. This would seem to be a unique adaptation within the ixodid lineage, even though cement-like proteins are found in argasid salivary gland transcriptomes [50]. The possibility therefore exists that cement might be secreted by argasid and *N. namaqua* larvae, which due to their small size has not been observed yet.

11–12) Rapid feeding in the nymphal and female stages occur in argasids and *N. namaqua* and were ancestral for ticks. The ancestral mode of dealing with excess water from the blood meal, was probably secretion via the Malpighian tubules as found in *N. namaqua* [5]. This function in argasids was taken over by the coxal organs [46]–[47]. However, coxal organs are generally found in the Acari, the holothyrida included, and would therefore have been ancestral [51]. Biological functions of coxal organs in the Acari are generally for the secretion of components involved in pre-oral digestion [51], and were probably lost in ixodids and *N. namaqua,* since secretion occurred via the salivary glands and Malpighian tubules. In the case of argasids their general increase in size might have led to the evolution of the coxal organs as a secretory system, as a more efficient mechanism of secretion compared to the Malpighian tubules.

13) During rapid feeding the blood meal ingested rarely exceeds 10-fold the body mass and is accompanied by little initial red blood cell lysis and slow digestion of the blood meal [47]. This leads to the requirement for several nymphal molts and the female feeding more than once, laying small egg batches between feeding events. This lifestyle was retained in argasids and may be associated with the “argasid-like” morphology (leathery cuticle) of nymphal and adult ticks in the ancestral lineage [3], [47]. In species of the *Antricola*, *Nothoaspis*, *Subparmatus* and *Otobius* sub-genera only larvae or nymphs require a blood-meal, while adults do not feed on vertebrates [43]. This trait is considered derived and could be lifestyle dependent and led to a significant reduction in salivary gland proteins involved in the regulation of the hosts defence mechanisms [52].

14–15) From this reconstruction it is clear that lifestyle functions unique to either ixodid or argasid lineages were present in the ancestral lineage. Specific evolution of these traits in the soft and hard tick families were therefore not required, but by exaptation were modified to suit specific lineage lifestyles. In the case of argasids, their lifestyle resemble largely that of the ancestral lineage, i.e. targeting vertebrates that frequent burrows or return to specific habitats [53]. This lifestyle accommodates shorter feeding events and the concomitant smaller egg batches, since progeny are guaranteed a blood-meal. In the case of ixodids, targeting of vertebrates without permanent burrows necessitated questing behaviour and strategies for optimal survival [53]. It was estimated that less than 1% of ixodid progeny complete their lifecycle because they fail to encounter hosts [54]. Ixodids therefore show a "type r" reproductive strategy, i.e. a high reproductive capacity to counteract low individual survival [54]. By exaptation of the prolonged engorgement phase from larvae to nymphs and adults, ixodids reduced the number of nymphal stages and the number of feeding events (host encounters) required to lay the numbers of eggs necessary to ensure survival of a fraction of the population. In the extreme form, this resulted in the evolution from a three-host to one-host life-cycle as observed in the genus *Rhipicephalus* (*Boophilus*) [53]. The evolution of ticks would therefore not have been necessarily shaped by host specificity but also by host and vector ecology [20], [23].

16) The presence of prolonged and rapid feeding capabilities in the ancestral lineage explains why ticks are the only blood-feeding arthropods with lineages that present both feeding modes. All other haematophagous arthropods feed rapidly [55]. This raises the question why ticks have evolved both feeding modes. The sister-group to the ticks is the holothyrida, a group of free living arthropod scavengers [56]. It has been assumed that ticks shared this lifestyle strategy before evolving blood-feeding behaviour [56]. It was also considered that the ancestral lineage fed on lymphatic fluids of vertebrate hosts [4] and this feeding stage could have resulted in the long feeding periods observed in ancestral larvae. It is likely that ticks lost the ability to synthesize heme during this period and became obligate blood-feeding parasites [57]. This in turn necessitated uptake of larger blood meals, which was initially accomplished by rapid feeding in nymphal and adult ticks. The lack of heme synthesizing capability has only been confirmed for ixodid ticks [57]. However, all ticks utilize vitellogenin as heme carrier protein, a feature unique to the Ixodida [57]. It′s therefore possible that loss of heme synthesizing capability and mechanisms to deal with heme toxicity occurred in the ancestral tick lineage.

The presence of shared characters in the *Nuttalliella* lineage that defines the major tick families is similar to that observed for stem groups, i.e. the presence of mixed characters found in extant lineages [58]–[59]. With the basal grouping of *N. namaqua* to the other tick families, this suggests that *N. namaqua* is not only the closest extant relative to the last common ancestral tick lineage, but may be an extant member of the stem group. This again supports the notion that this tick should be considered as a “living fossil” [5].

### Conclusions

In conclusion, nuclear and mitochondrial markers confirm that *N. namaqua* groups basal to both tick families. Molecular clock estimates indicated an origin of ticks in the Carboniferous (319 MYA). Many features associated with the phylogeography and host preference of extant ticks supports this origin. Phylogenetic mapping of various features associated with blood-feeding behaviour suggests that both the prolonged feeding periods associated with ixodid feeding and the short feeding of argasids were present in the ancestral tick lineage. In addition, a number of characters unique to argasid or ixodid ticks are found in *N. namaqua*. This suggests that *N. namaqua* is an extant stem group lineage, and that its status as “living–fossil” remains valid.

## Materials and Methods

### Ethics statement

All experiments related to lizard or mice feedings were performed in strict accordance with the Ethics guidelines from the Onderstepoort Veterinary Institute. Experiments were approved by the Onderstepoort Veterinary Institute Animal Ethics Committee (approval number: AEC12.11) and falls under the tick feeding and colony maintenance project. All necessary *Nuttalliella* collection and transport permits were obtained from the Veterinary Authorities (Permit number: SP2011/02/02/01).

### Tick collections

The *N. namaqua* specimens used in the current study were collected as previously described [5]. All necessary collection and transport permits were obtained from the Veterinary Authorities (Permit number: SP2011/02/02/01). In addition permission to collect ticks from Krymekaar and Voëlklip was granted by the owner, Mr. A. van Heerden. The *A. africolumbae* specimen was submitted to Onderstepoort for identification by Dr. Janet Mans, after this tick was found wandering among documents on the desk in her office at the Basic Medical Sciences Building, University of Pretoria, Pretoria. This building is the roosting site of numerous Speckled Rock pigeons and the tick was identified as a female *A. africolumbae* based on its original description [15], the fact that it was collected in the proximity of the original collection site and that it is the only member of the subgenus *Argas* thus far identified in South Africa.

### Next generation sequencing of tick genomic DNA

Genomic DNA was extracted as previously described [5]. Genomic DNA from single ticks (*N. namaqua*−40 ng, *A. africolumbae*−344 ng) was submitted to the Biotechnology Platform Next Generation Sequencing Service of the Agricultural Research Council (South Africa). Respectively, 26 and 55 ng of genomic DNA, for *N. namaqua* and *A. africolumbae,* was used for library preparation using the Nextera DNA sample preparation kit (Epicentre). Final prepared libraries were purified with the MinElute PCR purification kit (Qiagen) and quantified using a Qubit 2.0 fluorometer (Invitrogen). Sequencing was performed using Illumina V3 sequencing reagents and a mixture of Illumina and Nextera sequencing primers on the Illumina HiScanSQ sequencing machine. Single end sequencing reads of length 97 bp were generated, adapter sequences were trimmed and the last 19 bp removed before analysis. Reads were *de novo* assembled using the CLC Genomics Workbench v5.1 software package (mismatch cost-2, insertion cost-3, deletion cost-3, length fraction-0.5, similarity-0.95, minimum contig length-300, word size-50). The contigs obtained was formatted as a BLAST database and contigs coding for mitochondrial DNA were identified by TBLASTN analysis using the mitochondrial proteins from *C. capensis* and *I. hexagonus* as query sequences (Genbank accession numbers: 39653748, 5835680) [16], [18]. For *N. namaqua* 22,452,825 (1.7 Gb) reads assembled into 7,305 contigs (6,760,983 reads). Of these two large contigs (Contig_755: 9,219 bp, 4,605 reads, 39.3 average coverage and Contig_752: 5,336 bp, 2,266 reads, 33.5 average coverage) were identified that covered 100% of the full-length mitochondrial genome with a final size of 14,431 bp (Genbank accession number: JQ665719). Overlaps between the contigs occurred within the COIII and ND6 genes, so that no ambiguity existed with regard to the gene order of the mitochondrial genome. For *A. africolumbae* 11,248,267 (0.88 Gb) reads assembled into 7,207 contigs (2,454,572 reads). One contig (Contig_7196: 14,528 bp, 52,074 reads, 280 average coverage) covered 100% of the full-length mitochondrial genome with a final size of 14,440 bp (Genbank accession number: JQ665720). Open reading frames for different mitochondrial proteins were identified by translation of DNA sequences using the invertebrate mitochondrial code (code 5). Non-coding regions that code for tRNAs were identified using the ARWEN server [60], or by global alignment to the mitochondrial genomes of *O. moubata*, *O. porcinus*, *C. capensis*, *I. persulcatus* and *I. hexagonus*. Ribosomal RNA (16S and 12S) and complementary loop regions were identified by BLAST analysis. The final assembled genome was used as a scaffold to map reads back to determine coverage using the CLC Genomics Workbench v5.1 software package (mismatch cost-2, insertion cost-3, deletion cost-3, length fraction-0.5, similarity-0.95). A total of 6,868 and 52,072 reads were mapped to the *N. namaqua* and *A. africolumbae* mitochondrial genomes giving ∼37 and ∼285 fold average coverage, respectively.

### Phylogenetic analysis using the 16S rRNA gene

Soft tick sequences for the 16S rRNA gene was extracted from Genbank, as well as the sequences for *N. namaqua* and Allothyrida that was used as outgroups to give 44 sequences. Sequences were aligned using ClustalX [61], which yielded 299 parsimony informative sites. Maximum parsimony analysis was performed using MEGA5 [62]. Gaps were treated as a fifth character using partial deletion and a site coverage of 80%, trees were searched using Close-Neighbor-Interchange with ten random initial trees and ten thousand bootstraps were performed to give a bootstrap 50% consensus tree.

### Phylogenetic analysis using the 18S rRNA gene

The 18S rRNA gene for *A. africolumbae* was amplified, cloned and sequenced as previously described [5]. Other tick, holothyrid and opilioacarid sequences were extracted from Genbank. Sequences were aligned using a consideration of the secondary structure of RNA (Q-INS-i) as implemented in MAFFT [63]. Alignments were manually inspected, adjusted, edges trimmed, gapped positions and invariant sites removed to give 63 sequences with 273 phylogenetic informative sites. Bayesian analysis was performed using MrBayes 3.1.2 [64], using a General Time Reversible (GTR) model of nucleotide substitution with a proportion of invariant sites and a gamma distribution of among site heterogeneity using the nst = 6 rates = invgamma command. Four categories were used to approximate the gamma distribution and two runs were performed simultaneously, each with four Markov chains (one cold, three heated), which ran for 3,000,000 generations. The first 2,000,000 generations were discarded from the analysis (burnin) and every 100^th^ tree was sampled to calculate a 50% majority-rule consensus tree. Nodal values represent the posterior probability that the recovered clades exist, given the sequence dataset and are considered significant above 95% [27].

### Bayesian analysis of mitochondrial protein sequences

Sequences for the COI, Cytochrome b, ND1, ND2 and ND4 genes were extracted from the available arachnid mitochondrial genomes in GenBank. Multiple sequence alignment for each protein was performed separately using ClustalX [61]. All ambiguous sites (positions with gaps) were removed and alignments were concatenated to produce a supermatrix that existed of 1561 positions of which 1228 were variable (i.e. phylogenetically informative). A maximum likelihood search was performed to find the amino acid substitution model that fits the dataset the best using MEGA5 [62]. The MtREV24 +G +I model gave the lowest Bayesian information criterion (BIC) score (195023, range: 189933–225340) while using the lowest number of parameters (121, range: 119–140) with gamma shape parameter of 0.82.

Bayesian analysis was performed using MrBayes 3.1.2 [64], using the MtRev model of amino acid substitution with a proportion of invariant sites and a gamma distribution (Gamma factor = 0.82) of among site heterogeneity using the nst = 6 rates = invgamma command. Two runs were performed simultaneously, each with four Markov chains (one cold, three heated) which ran for 3,000,000 generations. The split frequencies were analysed to determine where the data converge and were used to determine the burnin at 1,000,000 generations. Every 100^th^ tree was sampled to calculate a 50% majority-rule consensus tree from 40002 trees analysed. Nodal values represent the posterior probability that the recovered clades exist, given the sequence dataset and are considered significant above 95% [27].

### Molecular clock estimation

Molecular clock estimates were performed using Bayesian analysis with PhyloBayes 3.3b [65]. The multiple alignment and topology of the 50% consensus tree obtained from the previous phylogenetic analysis of the mitochondrial genomes using MrBayes was used as input. The Xiphosura-Pycnogonida node (445±5 MYA) was used to calibrate the root of the tree, while minimum ages were assigned to the Ornithodorinae (94 MYA) and Metastriate (100 MYA) nodes [36]–[38], [44], [66]. *A. bituberculata* was specified as outgroup and a log-normal autocorrelated relaxed clock (-ln) was assumed, with a uniform prior on divergence times. A gamma prior of mean 2000 and standard deviation of 2000 million years were specified for the age of the root. Two runs were done for each analysis to determine that convergence occurred and for each run approximately 38000 cycles were run of which the first 2500 were discarded for the analysis. Dates used for Periods and Epochs are from the most recent Geologic Time Scale [67].

### Tick feeding on lizards and mice

Larvae were either fed on skinks (*Mabuya* genus) captured at Onderstepoort Veterinary Institute or Balb/c mice. Larvae were manually placed on animals and allowed to attach before animals were placed in their cages and monitored until larvae dropped. For proof of principle that larvae secrete water via their salivary glands, ticks were prefed on mice for three days, after which mice were injected intravenously with 100 mg/kg bromophenol blue in phosphate buffered saline solution. Larvae were removed after another five hours of feeding or were allowed to detach.
