# Correction: The Mitochondrial Genomes of *Nuttalliella namaqua* (Ixodoidea: Nuttalliellidae) and *Argas africolumbae* (Ixodoidae: Argasidae): Estimation of Divergence Dates for the Major Tick Lineages and Reconstruction of Ancestral Blood-Feeding Characters

**DOI:** 10.1371/annotation/19fe1c45-57c3-4008-9733-ebdf39202075

**Published:** 2013-07-31

**Authors:** Ben J. Mans, Daniel de Klerk, Ronel Pienaar, Minique H. de Castro, Abdalla A. Latif

There is a value that was incorrectly stated in the main text and several values that were incorrectly stated in Table 1. Please see the corrections to these values in the document at the following link:

http://www.plosone.org/corrections/pone.0049461.001.cn.pdf

